# Artificial Light at Night Advances the Onset of Vocal Activity in Both Male and Female Great Tits During the Breeding Season, While Noise Pollution Has Less Impact and Only in Females

**DOI:** 10.3390/ani14223199

**Published:** 2024-11-07

**Authors:** Kim Foppen, Rianne Pinxten, Marjolein Meijdam, Marcel Eens

**Affiliations:** Behavioural Ecology and Ecophysiology Group, Department of Biology, University of Antwerp, Universiteitsplein 1, 2610 Wilrijk, Belgium; annie.pinxten@uantwerpen.be (R.P.); marjolein.meijdam@uantwerpen.be (M.M.)

**Keywords:** ALAN, birdsong, chronotype, circadian rhythm, dawn chorus, emergence time, female vocalisations, noise pollution, *Parus major*

## Abstract

Artificial light at night (ALAN) and noise pollution are two important stressors associated with urbanisation that can have a profound impact on animal behaviour and physiology, potentially disrupting biological rhythms. Although the influence of ALAN and noise pollution on daily activity patterns of songbirds has been clearly demonstrated, studies often focus on males, and the few that examined females have not included the potential influence of males on female activity patterns. Using free-living pairs of great tits (*Parus major*) as a model, we examined for the first time the effects of ALAN and noise pollution and their interaction on the onset of (vocal) activity in both members of a pair. We focused on the egg-laying phase, when both sexes are most vocally active. The onset of male dawn song, female emergence time from the nest box and the onset of female calling in the nest box were measured and used as a proxy for the chronotype. The repeatabilities for all chronotype proxies were high, with higher repeatabilities for males. Consistent with previous studies, ALAN advanced the onset of male dawn song, while it did not elicit a strong response in female emergence time. Additionally, our results suggest an indirect effect of ALAN on the onset of female vocal activity via acoustic interaction with the male. Noise pollution advanced the emergence time in females, while an interaction between ALAN and noise pollution was found for the onset of female calling. In agreement with previous studies, several covariables were shown to have an influence on the activity onset. Taking several proxies for chronotype into account, this study has provided robust evidence of effects of ALAN on male and female cavity-nesting songbirds during the egg-laying period.

## Simple Summary

Urban environments often expose wildlife to artificial light at night and noise pollution, both of which can disrupt natural behaviours and daily rhythms. These disruptions can affect the health and survival of animals. In this study, we investigated how artificial light and noise affect the daily activity patterns of great tits, a common songbird species. We focused on both male and female birds during the egg-laying period, a critical time when they are most active. We measured when males began singing at dawn, when females left the nest, and when females started calling inside the nest. Our results showed that artificial light caused males to sing earlier in the morning but had less effect on females. However, females seemed to respond indirectly to artificial light through vocal interactions with their males. Noise pollution made females leave their nest box earlier, and the combination of light and noise affected the onset of calling in females. This research helps us better understand how urban stressors impact male and female birds differently, offering insights that could guide conservation efforts to protect wildlife in cities.

## 1. Introduction

Over the past three centuries, humans have become the dominant driver of patterns in global biogeochemistry and ecosystems, marking a new geological era called “The Anthropocene” [1,2]. Many human impacts during the Anthropocene are associated with a process called “urbanisation”, indicating the exacerbated growth of urban areas [2,3]. Most species living in urban areas are exposed to other ecological conditions and are confronted with different stressors than conspecifics living in natural conditions [4,5].

Artificial light at night (ALAN) and noise pollution are key urban stressors that can negatively affect the behaviour and physiology of urban-dwelling individuals, though their impact varies across species [6,7,8,9,10,11,12]. In addition to influencing orientation, movement, navigation [8,13,14,15,16], resource detection, predator avoidance [9,17] and communication [12,16], ALAN and noise pollution also disrupt biological rhythms [7,12,16,18].

Biological rhythms are based on daily, seasonal or tidal cycles that provide environmental cues to an endogenous system called the biological clock [19]. The biological clock provides organisms with an internal representation of time, which can be altered by environmental cues and results in the expression of specific periodic behaviours and physiologies [19]. The phase and period length of the biological clock vary substantially among different individuals, even when exposed to the same environmental cues, and can, therefore, be subjected to natural and sexual selection [19,20,21]. A high consistency in individual timing gives individuals a certain temporal phenotype or chronotype [19].

One of the key regulators of daily rhythms in terrestrial vertebrates is the day–night cycle. Changes in light conditions, such as those caused by ALAN, affect sleep–wake cycles and daily rhythms by altering melatonin production [22,23,24,25]. Numerous studies on songbirds, conducted in both wild and captive settings, using experimental and correlational designs, have demonstrated that ALAN consistently advances the onset of activity in songbirds [4,7,24,26,27,28,29,30,31,32,33,34]. Because measuring chronotypes in the wild is challenging, proxies like activity onset (e.g., dawn song in males) and offset (e.g., dusk chorus) are commonly used. In females, emergence time from a nest (e.g., nest box) in the morning serves as a proxy.

While noise pollution has also been shown to affect sleep–wake cycles [35], its effects on melatonin are less understood and more inconsistent across studies [6,32,36,37,38,39]. Since ALAN and noise pollution often co-occur in urban areas, their interaction has recently gained attention, with noise appearing to amplify the effects of ALAN [38,40,41,42,43].

Most studies have only examined the influence of ALAN and noise pollution on daily rhythms of male songbirds (e.g., [4,6,23,24,26,27,29,32,33,34,36,37,38,39]), while fewer studies have looked at their influence on daily rhythms of female songbirds (but see [7,31,44,45,46,47]). As sex-specific differences in activity patterns have been found in several songbird species [45,46,47], males and females may be affected differently when being exposed to ALAN or noise pollution, especially during the breeding season (e.g., [41]).

Moreover, males and females may experience different (levels of these) stressors, particularly in cavity-nesting species. Females often sleep inside cavities during the breeding season, which can shield them from direct ALAN exposure, as demonstrated in great tits (*Parus major*) by Raap et al. [48]. In contrast, males, who often sleep outside cavities during this period, are more likely exposed to ALAN. Experimental studies have shown that female emergence time advances when exposed to ALAN inside nest boxes [7,31,44], but it remains unclear if free-living females exposed to ALAN from the surrounding area are affected in a similar way.

Additionally, (vocal) interactions between the members of a pair might partly explain intra-individual variation in the onset of activity during the breeding season. In several cavity-nesting species, including the great tit, pairs engage in extensive vocal communication before and during the egg-laying phase. Females often call from the nest box in response to male vocalisations, indicating that they are awake before emerging [49,50,51,52,53]. This communication could allow males to influence the chronotype of their mates, suggesting that light and noise pollution experienced by males may indirectly affect female activity onset. These examples highlight the need to consider both pair members when assessing the effects of urban stressors on daily rhythms.

In the present study, we investigated the effects of ALAN and noise pollution and their interaction on the activity onset of both pair members in free-living suburban great tits during the egg-laying phase of the breeding season. Besides the onset of male dawn song and female emergence time, the onset of female calling behaviour was used as a proxy for the activity onset of the female. Covariables that could potentially have influenced activity onset, such as ambient temperature [4,45,54,55], precipitation [45] and the stage of the egg-laying phase [45,46,47,52], were also taken into account. Using this approach, we not only investigated direct effects of anthropogenic stressors on male and female great tits but also potential indirect effects on females that were transmitted by the male via vocal communication. This has, to the best of our knowledge, never been conducted before. Finally, given that repeated measurements of the onset of vocal activity were obtained in a large number of individuals for both sexes, we also determined the repeatability of these behaviours.

## 2. Materials and Methods

### 2.1. Study Species, Study Area and General Procedures

The great tit was used as a model species in this study. It is a relatively short-lived, socially monogamous, cavity-nesting songbird species that easily breeds in artificial nest boxes and inhabits urban areas, making it an important model species in evolutionary and environmental research [56,57]. Great tits can be observed and captured year-round throughout their entire life, and, since they are highly site-faithful, the environmental stressors they are exposed to can be controlled for or manipulated. Data were collected during the breeding season of 2021 (between March and June) in a resident suburban nest-box population of great tits at the university campus Drie Eiken in Wilrijk, Antwerp (Figure S1), Belgium (51°9′44″ N, 4°24′15″ E). The study area contains around 170 nest boxes, and the population had been monitored for more than 20 years, with all individuals being (colour) ringed, provided with Passive Integrated Transponders (tracking tags), sexed and weighed every year (see, e.g., [44,58,59,60,61]). This allows for the gathering of detailed information on individuals in the population. The nest boxes were made out of plywood with a metal ceiling and a round opening of 30 mm (see [58]). During the breeding season, all occupied nest boxes were checked on a daily basis to determine the start and progression of the egg-laying phase and the clutch size.

### 2.2. Recording and Assessment of the Onset of Activity

The onset of male dawn song, the onset of female calling and female emergence time were measured using SongMeters (SongMeterTM SM2+; Wildlife Acoustics, Inc, Maynard, MA, USA). A SongMeter is an acoustic monitoring and data-logging device that can be programmed to record automatically on a schedule. It was placed on the roof of a nest box and connected to a microphone on the outside (to record male dawn song) and a small microphone on the inside of nest box (to record female calling). Both microphones were used to assess female emergence time. At the start of egg-laying, female fertility peaks, resulting in vigorous male dawn chorus and vocal communication before the female emerges, while both decrease as the egg-laying phase progresses towards the incubation phase [52,62]. Therefore, a SongMeter was placed on the nest box on the first day of egg-laying, recording the onset of activity from the day of the second until the fifth egg (four mornings), after which it was removed to extract the data of the recordings. For a minority of the nest boxes, the SongMeter did not record the onset of activity for the full four-day period (second until fifth egg) but for two or three days within this period. The SongMeters were set to record from 3:00 until 8:00 CET. Sunrise during the breeding season falls within these time intervals, and the margins are large enough to ensure recording of the onset of activity, even in exceptionally early or late individuals. The placement or removal of the SongMeters was carried out several hours before sunset or after sunrise to minimise disturbance to the birds.

**Figure 1:**
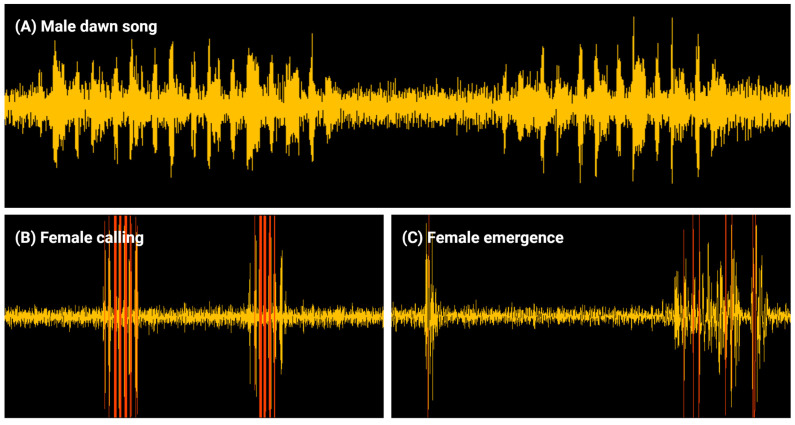
Sound waves of male dawn song, female calling and female emergence. Sound waves of the SongMeter recordings could be visualised using Avisoft-SASLab Pro (version 5.2.15) [63]. Typical patterns for male dawn song (**A**), female calling (**B**) and female emergence (**C**) are shown and were used to determine the onset of activity.

The software Avisoft-SASLab Pro (version 5.2.15) [63] was used to determine the proxies for activity onset recorded by the SongMeters (Figure 1). To assess the onset of male dawn song, the timing of the first call or song of the male was used (similar to Naguib et al. [55]; Figure 1A). If the dawn song of several males could be heard and seen on the spectrogram, the recording was only used if the onset of the song of the male occupying the nest box was clearly distinguishable from the other males. This was the case when the amplitude of the song of this male was significantly higher than that of the songs of the other males right from the onset of dawn song (indicating that this male was closest to the nest box) or when the amplitude of a recognisable song type was low at the onset of dawn song but increased after a certain time period (indicating that this male had moved closer to the nest box, which males typically do as the dawn chorus progresses). Although most females call from the nest box in response to their male before they emerge in the morning ([49,50,51,52,53]; Figure 1B), some females in our study did not call every morning, making it impossible to determine the onset of female calling for these mornings. Female emergence time could be determined for all recordings and was assessed using the sound of her claws on the entrance of the nest box on the recording from the microphone inside the nest box and the movement of her wings when taking off on the outside microphone (Figure 1C; also see [52]).

### 2.3. Measurements of ALAN and Noise Pollution

In our (sub)urban study area, there is a large variation in ALAN and noise pollution because of the presence or absence of parking lots, streetlights, a highway and roads (see [64,65]). For each occupied nest box, light intensity and noise amplitude at night (between 22:00 and 01:00) were measured a few days after the SongMeter had been removed from the nest box to avoid disturbance of the female in the nest box during the nights preceding the recording. Maximum light intensity (lux) was measured with an ILM 1335 light meter (ISO-TECH, Northamptonshire, UK). To get a proxy for the amount of ALAN experienced by the female inside the nest box, the light detector was placed vertically at the nest box opening. To get a proxy of the amount of ALAN experienced by the male, the light detector was pointed skywards and the average of five measurements (at the opening, one meter north, east, south and west) around the nest box was used (similar to [64]). The maximal noise amplitude (decibel) was measured by holding a sound (decibel) meter (DVM401, Velleman Inc., Fort Worth, TX, USA) approximately one meter from the nest box opening for a duration of 10 s. The highest value of noise amplitude (sound pressure levels (SPLs) relative to 20 μPa; dB; A-weighted; fast response) measured during these 10 s was noted. This measurement was repeated twice within 1–2 min. Noise measurements were made only in the absence of extreme sources of noise (e.g., lawn mowers and leaf blowers but including the consistent traffic flow on the highway) and, therefore, did not encompass peaks in noise levels but rather represented maximum levels of background noise (similar to [41,65]). An average of the two repeated measurements was used as a proxy for the amount of noise pollution the pair of great tits experienced at the particular nest box (similar to [64]). Since noise pollution can be relatively variable over short time periods, the noise amplitude was also measured during the day for a sample of 11 nest boxes.

### 2.4. Weather Variables

Recorded values (10 min intervals) of precipitation (mm) and global temperature (°C) were obtained from a reliable local weather station located ~3 km from the study area (coordinates: 51°10′05″ N, 4°23′39″ E) and accessed through Weather Underground (https://www.wunderground.com (accessed on 5 July 2021)). The temperature (°C) and precipitation (mm) around sunrise on the morning of the SongMeter recording were calculated by taking the average of the two values closest to sunrise. The lowest night temperature was extracted from values in the night preceding the SongMeter recording.

### 2.5. Statistical Analyses

The proxies for the onset of activity were all converted to times (min) relative to sunrise. Multiple SongMeter recordings during the egg-laying phase were obtained for 106 pairs. A pair was only included in the dataset when both the proxy for the male and the female could be determined from the SongMeter recordings, which was the case for 74 pairs (Figure S1). ALAN could not be determined for one of the females, resulting in a complete dataset for 74 males and 73 females (Table S1).

For all statistical analyses, R 4.1.1 [66] was used. To avoid convergence problems in the models, all predictors were centred and scaled before being used in the data analyses by subtracting the mean and dividing by the standard deviation [67]. For each activity onset proxy (onset of male dawn song, *n* = 268; female emergence time, *n* = 264; onset of female calling, *n* = 237), a separate linear mixed-effects analysis (LMER) was performed using the lme4 package (version 1.1-27.1) [68]. Based on the hypothesis, a basic model was constructed for each activity onset with the predictors ALAN (lux) and noise pollution (dB) and their two-way interaction as fixed effects. For noise pollution, night time noise levels were used, but the correlation between day- and night-time noise levels was determined using Spearman’s correlation coefficient. Nest box ID and Julian date (number of days since 31 March) were included as random effects (with random intercepts) to account for non-independence of observations from the same individual or on the same day. Since every individual in the dataset occupied only one nest box during the season, nest box ID equals bird ID.

During model selection a (partial) step-up strategy was used (see Table S2) by comparing the Akaike information criteria (AIC) and R^2^ (using the MuMIn package (version 1.43.17); [69]), in which potential covariables were assessed. Potential covariables were temperature around sunrise (°C), lowest night temperature (°C), precipitation around sunrise (mm) and time after the start of the egg-laying phase (days). In addition, the onset of male dawn song (min) could be included in the models for activity onset of the female since the onset of activity was measured individually for each bird in the population and the pairs occupying the nest boxes were known.

The final models obtained for the onset of male dawn song (min) and female emergence time (min) included ALAN (lux) and noise pollution (dB), as well as their two-way interaction, time after the start of the egg-laying phase (days) and lowest night temperature (°C) as fixed effects, while nest box ID and Julian date were added as random effects. For the onset of female calling (min), the onset of male dawn song (min) was added as a predictor in the final model as well. To account for the fact that the correlation between the onset of female calling and male dawn song had a cut-off point, a quadratic term was added for the onset of male dawn song. For significant interaction terms, the Johnson–Neyman procedure was used to calculate for which part of the range of the predictors the effect was significant (using the interactions package (version 1.1.6); [70]). We performed posterior predictive checks to ensure model fit, verified linearity, assessed homogeneity of variance, checked for normality of residuals and accessed multicollinearity (using the performance package (version 0.8.0); Figure S2) [71]. Outliers or influential observations in the models were detected using Cook’s distances (≥1 was considered influential). Several influential observations were detected in each of the models and the quantile–quantile plots of fixed and random effects of all models showed distributions with symmetrical heavy tails (Figure S2). To avoid inflation of the standard errors of the fixed and random effects, a robust fit of linear mixed-effects models was used by using the package robustlmm (version 2.4-4) [72]. The robust estimation method in robustlmm provides a way to deal with contamination by down-weighing observations with a large absolute value (Figure S3). Since the models with a robust fit had similar estimates as the models with a normal fit, but with smaller CI’s and considerably higher marginal R^2^ values (Figure S4; Tables 1 and S3), these estimates are used in the results, while the estimates of the models with a normal fit can be found in the Supplementary Materials.

One of the occupied nest boxes in the population was exposed to extremely high levels of ALAN. At this nest box, a value of 17.0 lux was measured, while all the other nest boxes had a value between 0.01 and 5.5 lux. To examine if the model outcomes (especially with regard to ALAN) were largely determined by the individuals of this nest box only, alternative (robust) linear mixed-effects models, excluding this particular pair of great tits, were constructed using the same model-selection methods and compared to the models with a full dataset (Table S3).

For the models with a normal fit, the adjusted repeatability (i.e., repeatability of the onset of activity corrected for fixed effects in the models) for the random-factor nest box ID (=bird ID) was calculated (using the rptR package (version 0.9.22); [73]), and bootstrapping was used to provide 95% confidence intervals (CIs).

**Table 1:** Statistical output of robust mixed-effects models for the onset of activity. For each proxy for the onset of activity, the fixed-effects part of the table shows the regression estimates (with standard error) in minutes, 95% confidence intervals (CI 95%), t-values and *p*-values (Satterthwaite approximation). All predictors are standardised, meaning that for each standard deviation increase in the predictor, the change in the onset of activity (in minutes) is given by the estimate. Significant values (*p* < 0.05) are depicted in bold. In the random-effects part of the table variances of both random factors are shown (τ00), as well as the residual variance (σ^2^) and the intraclass correlation coefficient (ICC). The marginal and conditional R^2^ for the robust mixed-effects models are calculated using the robustlmm package [72] according to Nakagawa and Schielzeth [74].

	*Onset Male Dawn Song*				*Female Emergence Time*				*Onset Female Calling*			
Predictors	Estimates	CI (95%)	T-Value	*p*-Value	Estimates	CI (95%)	T-Value	*p*-Value	Estimates	CI (95%)	T-Value	*p*-Value
*(Intercept)*	−46.88 (2.24)	−51.28–−42.49	−20.92	**<0.001**	12.53 (1.76)	9.08–15.98	7.12	**<0.001**	−21.04 (2.17)	−25.30–−16.78	−9.68	**<0.001**
*ALAN*	−8.44 (1.81)	−11.99–−4.90	−4.67	**0.001**	−3.04 (1.13)	−5.26–−0.82	−2.68	**0.024**	−4.30 (1.87)	−7.96–−0.65	−2.30	**0.011**
*Noise pollution*	−1.41 (2.18)	−5.69–−2.87	−0.65	0.860	−2.67 (1.34)	−5.29–−0.05	−2.00	**0.027**	0.13 (1.90)	−3.60–3.85	0.07	0.956
*Lowest night temperature*	−1.33 (0.68)	−2.65–−0.00	−1.96	**0.045**	−3.42 (1.30)	−5.95–−0.88	−2.64	**0.022**	−2.17 (0.95)	−4.04–−0.31	−2.29	0.086
*Days after first egg*	1.49 (0.53)	0.45–2.52	2.82	**0.003**	1.21 (0.75)	−0.26–2.67	1.62	0.094	5.88 (1.03)	3.85–7.90	5.70	**<0.001**
*Onset male dawn song*									6.14 (2.44)	1.35–10.93	2.52	**0.017**
*Onset male dawn song²*									1.78 (1.01)	−0.20–3.76	1.76	0.098
*ALAN × noise pollution*	3.93 (3.51)	−2.94–10.81	1.12	0.244	−0.16 (1.58)	−3.26–2.94	−0.10	0.575	−6.12 (2.32)	−10.66–−1.58	−2.64	**0.015**
**Random effects**												
*σ²*	20.09				41.11				114.39			
*τ00 nest box ID*	317.30				100.62				198.04			
*τ00 Julian date*	5.54				28.72				0.00			
*ICC*	0.94				0.76				0.63			
*N nest box ID*	74				73				73			
*N Julian date*	26				25				24			
*Observations*	268				264				237			

## 3. Results

### 3.1. ALAN and Noise Pollution in the Study Area

**Figure 2:**
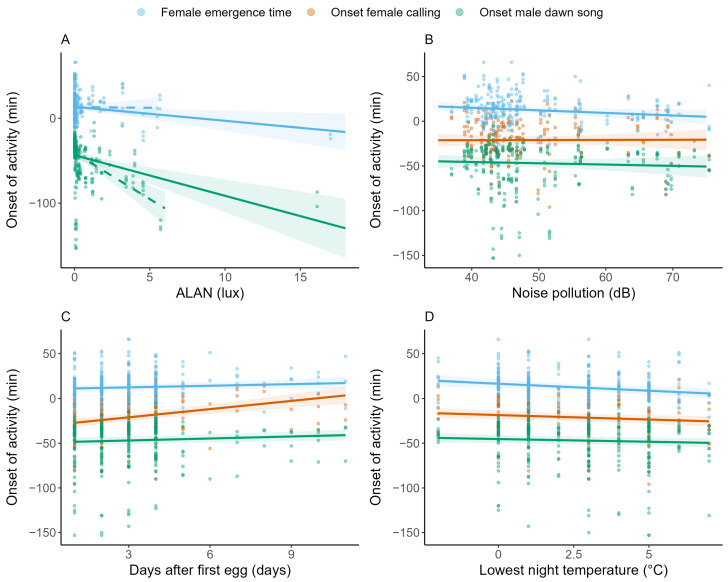
Effect of predictors on the onset of activity. For each proxy for the onset of activity (indicated by the different colours), a predicted regression slope (line) with 95% CI (shaded area) for the models with a robust fit is shown for ALAN (**A**), noise pollution (**B**), the number of days after the first egg (**C**) and the lowest night temperature (**D**), while controlling for other predictors. The slope for ALAN for the onset of female calling is not illustrated because there was a significant interaction effect with noise pollution (see Figure 3). The slopes for ALAN for robust models with an alternative dataset are shown as well ((**A**); indicated with dashed lines). Raw data are plotted in the figures, and standardised predictors are back-transformed for interpretability.

Levels of ALAN measured varied between 0.01 and 16.12 lux around the nest box (mean = 0.70 lux; median = 0.08 lux) and 0.01 and 17.00 lux at the entrance hole of the nest box (mean = 0.59 lux; median = 0.01 lux), with 95% of the measurements between 0.01 and 4.60 lux and 0.01 and 5.49 lux, respectively (Figure 2A). Levels of noise pollution at night varied between 37.00 and 75.40 dB, with a mean of 49.16 dB and a median of 45.50 dB (Figure 2B). There was a strong positive correlation between day- and night-time noise levels (Spearman’s correlation coefficient = 0.64).

### 3.2. Effect of ALAN, Noise Pollution and Covariables on the Onset of Activity

#### 3.2.1. Onset of Male Dawn Song

Male great tits started their dawn song on average 46.88 min (SE = 2.24, CI = −51.28–−42.49) before sunrise, when controlling for all predictors (Figure 2; Table 1). The onset of male dawn song was significantly affected by ALAN (t = −4.67, *p* = 0.001), the lowest night temperature (t = −1.96, *p* = 0.045) and the number of days after the female laid her first egg (t = 2.82, *p* = 0.003). Noise pollution and the interaction between ALAN and noise pollution did not significantly affect the onset of male dawn song (Table 1). ALAN strongly advanced the onset of male dawn song, with males starting their dawn song on average 8.44 min (SE = 1.81, CI = −11.99–−4.90) earlier per standard deviation (=1.77 lux) increase in ALAN (Figure 2A; Table 1). For the lowest night temperature and the amount of days after the female’s first egg, the effect sizes were small but consistent, with a 1.33 min (SE = 0.68, CI = −2.65–0) advancement in the onset of male dawn song per standard deviation increase in the lowest night temperature (=2.18 °C) and a 1.49 min (SE = 0.53, CI = 0.45–2.52) delay per standard deviation increase in the number of days after the female’s first egg (=2 days; Figure 2C,D; Table 1). The proportion of the total variance explained by the fixed effects was moderate (marginal R^2^ = 0.19; [75]). When the male of the most light-polluted nest box was excluded from the dataset, the onset of male dawn song was still significantly influenced by ALAN (t = −6.22, *p* = <0.001) and advanced with 19.19 min (SE = 3.08, CI = −25.04–−13.15) per standard deviation (=1.16 lux) increase in ALAN (Figure 2A; Tables 1 and S3A).

#### 3.2.2. Female Emergence Time

When controlling for all predictors, female emergence time was on average 12.53 min after sunrise (SE = 1.76, CI = 9.08–15.98; Figure 2; Table 1). ALAN significantly (t = −2.68, *p* = 0.024) advanced female emergence time (−3.04 min, SE = 1.13, CI = −5.26–−0.82) for every standard deviation increase in ALAN (1.84 lux; Figure 2A; Table 1). However, when the female from the most light-polluted nest box was removed from the dataset, this significant effect disappeared (−0.29 min, SE = 2.18, CI = −4.56–3.97; Figure 2A; Table S3B). Noise pollution significantly (t = −2.00, *p* = 0.027) affected female emergence time, with an advancement of 2.67 min (SE = 1.34, CI = −5.29–−0.05) for every standard deviation increase in noise pollution (9.35 dB; Figure 2B; Table 1). There was no significant interaction between ALAN and noise pollution (Table 1). Contrary to the effect on the onset of male dawn song, the number of days after the female’s first egg did not significantly delay her emergence time. Female emergence time did advance significantly (−3.42 min, t = −2.64, *p* = 0.022) with every standard deviation increase in the lowest night temperature (2.19 °C), when controlling for all other predictors (Figure 2D; Table 1). The proportion of variance explained by the fixed effects was moderate (marginal R^2^ = 0.16; Table 1).

#### 3.2.3. Onset of Female Calling

**Figure 3:**
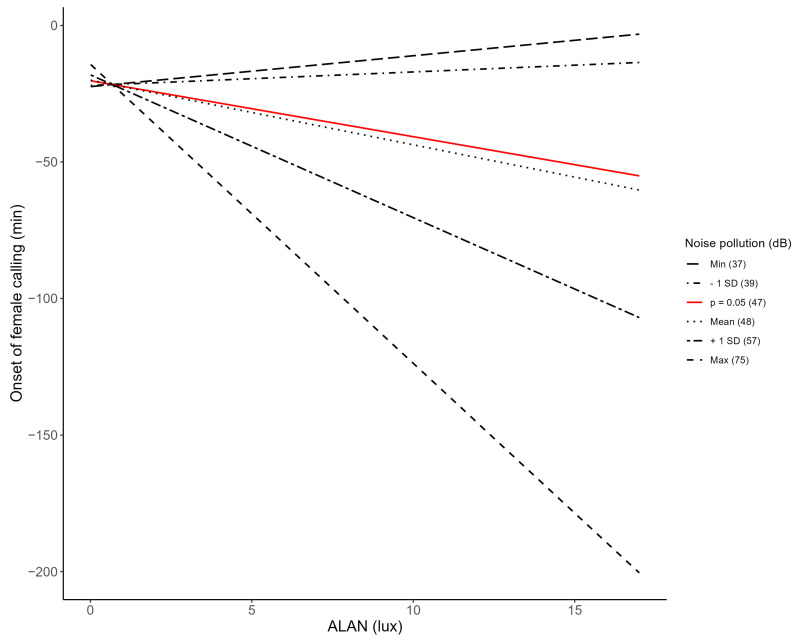
Interaction effect of ALAN and noise pollution on the onset of female calling. For the effect of ALAN on the onset of female calling, predicted regression slopes of the model with a robust fit are shown. The regression slopes for several indicative values of noise pollution are shown for illustrative purposes, including the slope in red for which *p* = 0.05 (based on Johnson–Neyman plot; Figure S6). Slopes above the red slope do not significantly differ from zero, while slopes below do.

**Figure 4:**
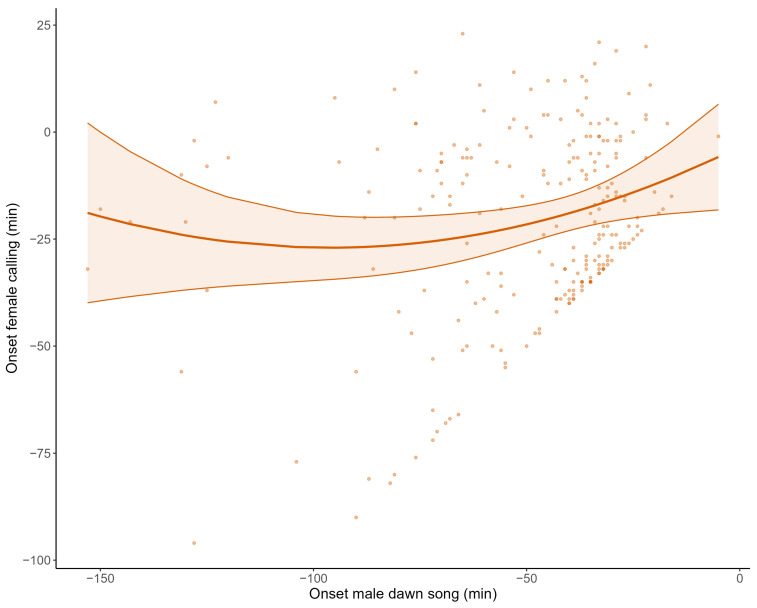
Effect of the onset of male dawn song on the onset of female calling. For the model of the onset of female calling with a robust fit, the predicted regression slope (line) with 95% CI (shaded area) is shown for the onset of male dawn song while controlling for other predictors. A quadratic term (although not significant) was added to explain the curvature in the relationship. Raw data are plotted in the figures, and standardised predictors are back-transformed for interpretability.

Female great tits started calling on average 21.04 min before sunrise (SE = 2.17, CI = −25.30–−16.78), when controlling for all predictors (Figure 2; Table 1). There was a significant interaction effect between ALAN and noise pollution on the onset of female calling (t = −2.64, *p* = 0.015; Table 1). For females from a nest box with high levels of noise pollution, ALAN significantly advanced the onset of calling, while the Johnson–Neyman procedure shows it was not significant anymore for females from a nest box with noise pollution levels of 47 dB or lower (Figure 3 and Figure S6). The (linear term of the) onset of male dawn song significantly influenced the onset of female calling (t = −2.51, *p* = 0.015; Table 1), with females delaying their onset of calling by 6.14 min (SE = 2.44, CI = 1.35–10.93) per standard deviation delay (26.51 min) of the onset of male dawn song (Figure 4).

Females never started calling before the onset of dawn song of their male (pers. obs.), and, only from 100 min before sunrise onwards, there were females that responded immediately to the onset of the dawn song of their male. Due to this cut-off point, the linear effect alone did not adequately capture the relationship between the onset of male dawn song and female calling, so a quadratic term was necessary to explain the curvature in the relationship. However, the model output shows that the quadratic effect did not significantly contribute to explaining the variation in the response variable beyond what the linear term already captured. Contrary to the onset of male dawn song and female emergence time, the onset of female calling was not significantly affected by the lowest night temperature, while the number of days after the female laid her first egg strongly (t = 5.91, *p* = <0.001) delayed the onset of female calling behaviour (6.22 min, SE = 1.05, CI = 4.15–8.28; Table 1; Figure 2C) per standard deviation (1.92 days). When the pair of great tits from the most light-polluted nest box was removed, ALAN significantly delayed the onset of calling for females from nest boxes with noise pollution levels of 51 dB or higher, while the effect of the other predictors did not significantly change (Figures S5 and S6). The proportion of variance explained by the fixed effects was high (marginal R^2^ = 0.31; Table 1).

### 3.3. Repeatability of Onset of Activity

For all models, the proportion of the variance explained by both the fixed and the random effects was very large, with a conditional R^2^ of 0.95 for the robust model of the onset of male dawn song, 0.80 for the female emergence time and 0.75 for the onset of female calling (Table 1). Most of this variance (τ00) could be explained by differences among individuals (i.e., nest box ID). For the models with a normal fit, the adjusted repeatability (i.e., repeatability of the onset of activity corrected for fixed effects in the models) of each proxy for the onset of activity was calculated. The repeatability for the onset of male dawn song was very high (0.94, CI = 0.92–0.96). The repeatability for female emergence time was relatively high as well (0.54, CI = 0.41–0.66), while it was moderate for the onset of female calling (0.43, CI = 0.30–0.57).

## 4. Discussion

Using a correlational approach involving a large number of free-living pairs of great tits, we examined for the first time the effects of ALAN and noise pollution and their interaction on the onset of (vocal) activity of both members of a pair during the egg-laying phase, including the potential influence of males on the activity pattern of their female. ALAN greatly advanced the onset of dawn song in males, while it only advanced female emergence time when the extremely light-polluted nest box was taken into account. Furthermore, an interaction effect between ALAN and noise pollution was found only for the onset of female calling. The results suggest that there was a small effect of noise pollution on the onset of female, but not male, activity. Additionally, in agreement with previous studies, several covariables were shown to influence the activity onset, including the lowest night temperature and the number of days after the female had laid her first egg. The interpretation, possible underlying mechanisms and implications of these results are discussed below.

### 4.1. ALAN Has Direct and Indirect Effects on the Onset of Activity

Our observation that ALAN greatly advanced the onset of dawn song in male great tits aligns with previous studies in this and other temperate songbird species [4,27,32,33,34]. Notably, males in our suburban population began their dawn song approximately one hour earlier than those in rural forest areas (experimentally) exposed to ALAN [27,32,33,34]. This difference is consistent with previously observed disparities in activity onset between urban and forest-dwelling male songbirds, possibly linked to variations in circadian period length [26,38].

Furthermore, it is noteworthy that some males began singing early despite being exposed to low levels of ALAN. This phenomenon may be attributed to the possibility that urban populations have been selected for early chronotypes over generations of exposure to artificial lighting. As a result, even individuals not exposed to significant light at night may still exhibit a default early chronotype. Additionally, some males may actively approach street lights in the morning to begin their dawn song (pers. obs.). Moreover, we observed a cluster of early-singing males, some exposed to high levels of ALAN, while their neighbours, who also began singing early, were not. Previous studies have shown that neighbouring males can influence each other’s timing of dawn song [56,76,77], suggesting that males in brightly lit areas, who started their dawn song early, may have triggered nearby conspecifics from darker territories to begin singing as well.

The range of ALAN in our study population was 0.010–17.0 lux. However, no values were found between 5.5 and 17.0 lux because there were few nest boxes with high levels of ALAN, and several of these nest boxes were excluded from the dataset due to missing data. Despite this, our results suggest that the effect of ALAN on the onset of male dawn song was not solely driven by the pair of great tits from the most light-polluted nest box (17.0 lux). In fact, removing this pair from the dataset strengthened the observed effect of ALAN. Conversely, excluding the female from the same nest box eliminated the significant effect of ALAN on female emergence time. Previous studies indicated that light levels below 8.0 lux were unlikely to penetrate the bottom of nest boxes [48], which might explain the lack of an effect on female emergence in these conditions. To test whether light reached the bottom of the nest box at 17.0 lux, we exposed a nest box to this intensity and placed a sensor inside. The sensor indicated that some light did indeed penetrate to the bottom. Therefore, it seems likely that there is a threshold somewhere between 5.5 and 17.0 lux for light to reach the bottom of the nest box, suggesting that the female in the most light-polluted nest box was directly influenced by ALAN while the other females were not. Furthermore, the fact that egg-laying constrains the female’s emergence time during this phase (with great tit females laying an egg before leaving the nest box [62]) makes detecting a potential effect of ALAN on emergence time more difficult.

Due to the extensive vocal communication between pair members before the female emerges in the morning [49,50,51,52,53], males likely influence the timing of female calling. Our results indeed indicated that the onset of male dawn song influenced the onset of female calling. Females never began calling before their male’s onset of dawn song and always responded to his vocalisation (pers. obs.). Interestingly, females only responded immediately to the onset of the dawn song of their male from 80 min before sunrise onwards, suggesting that there is a lower time limit to the onset of calling. A possible explanation includes that females only call in response to their male when they are awake. Another possibility is that differences in the male’s singing position at the onset of dawn song play a role. Early males sometimes began singing farther from the nest box and approached it later in the morning (pers. obs.). This supports the hypothesis that some early males began their dawn song by approaching nearby light sources, thus starting farther from the nest box and out of the female’s hearing range. Since the onset of female calling is influenced by the onset of male dawn song, which in its turn is influenced by ALAN, our results suggest an indirect effect of ALAN on the onset of female calling via their male’s song. ALAN advanced the onset of female calling only for females from a nest box with relatively high levels of noise pollution. This interaction effect could also be explained by differences in the male’s singing position. When the environment was noisy, males often sang their dawn song in close proximity to or even on the nest box (pers. obs.). Females usually responded immediately to their male, which led to a strong association between the onset of female calling and the onset of male dawn song (and, therefore, ALAN) in noisy areas.

Although further research is recommended, our study is, to the best of our knowledge, the first to report an indirect effect of ALAN on females via communication with their males. It stresses the importance of taking intra-pair vocal communication into account when looking at the effects of urban stressors. When taking the onset of female calling as a proxy, the environmental factors influencing the female chronotype largely reflected the factors influencing the chronotype of her mate, which could have significant implications for understanding how urban stressors affect cavity-nesting females during the breeding season. Through a field experiment, Halfwerk et al. [78] have shown an indirect effect of noise pollution on the song behaviour of male great tits due to social feedback from the female. Similar field experiments like those performed by Halfwerk et al. [78], in which one individual of a pair is exposed to the stressor while the other is not, are recommended to further explore indirect effects of ALAN on songbirds.

### 4.2. Noise Pollution Only Affects the Onset of Activity in Females

In our study, we found that noise pollution did not affect the onset of male dawn song, aligning with the findings of Da Silva et al. [32]. However, our results contrast with several other studies on temperate songbirds [4,6,36,37,79,80], which indicated that elevated noise pollution levels were associated with an earlier onset of male dawn song. This response may serve as an adaptive mechanism to counteract the masking of acoustic signals. We propose several explanations for the absence of an effect of noise pollution in our study population. Firstly, most of these earlier studies did not look at the response to noise pollution at an individual level within a population (but see [4]). Adaptive changes in the onset of activity to noise pollution might be visible among different populations but not within a population. Dominoni et al. [38] exposed great tits to noise pollution in a within-individual experimental set-up. Although they found that anthropogenic noise reduced the amount of (daytime) activity, it did not advance the onset of activity neither in individuals from urban nor forest populations. This may suggest that the short-term plastic response of individual great tits to noise pollution is limited to changes in the amount of activity and not in the onset of activity. Additionally, it may be very costly in terms of sleep reduction to avoid the masking of acoustic information by advancing the onset of dawn song, especially in species with a relatively late onset of dawn song under natural conditions [4,44]. Vocal adjustments and increasing the proximity to the nest box when singing to minimise masking by noise pollution might be less costly solutions [78,81,82,83].

Contrarily, noise pollution did advance female emergence time, although the effect size was small. Noise pollution could influence females directly, as the earlier emergence time may indicate that the female has woken up sooner. Besides the implications of a reduction in sleep duration [41], such changes in behaviour could have ecological consequences, including an increased risk of predation from nocturnal or crepuscular predators [84], as well as higher energy expenditures due to greater exposure to colder temperatures [85]. Additionally, the impact of noise pollution on females may also be indirect. Although the male may have found ways to reduce masking of his acoustic signals, the female could still experience altered signal-to-noise ratios or struggle to determine his exact location from the nest box. This hypothesis is supported by a sound transmission experiment with great tit songs in nest boxes, which demonstrated possible signal distortion [86], and may compel a female to leave her nest box earlier.

### 4.3. Measurements of ALAN and Noise Pollution

We did not measure the levels of ALAN and noise pollution on the same days of the SongMeter recordings. We made this choice to avoid disturbing the nesting environment with our presence during the recordings. We believe this decision did not significantly influence our results, as previous measurements in our study area have consistently shown similar levels of both ALAN and noise pollution [41,65,87,88]. Consistent with measurements from the current study, 24 h recordings near nest boxes in our study population indicated that day- and night-time noise levels were highly correlated [41,87]. Furthermore, these recordings showed a high correlation between short timeframe and longer-term (24 h) measurements. This can be explained by the fact that the soundscape in the surroundings of the nest boxes is largely determined by the consistent noise generated by the E19 highway at the border of the study area [41,65]. In this study, we used background noise measurements because peak noise measurements can be highly variable and context-dependent, necessitating simultaneous measurements of SongMeter data and noise levels. Previous research in our study population has demonstrated that background noise levels, similar to peak noise levels, also influence sleep and daily rhythms in great tits [89]. By focusing on background noise, we could capture its consistent effects on daily rhythms without the disturbance of the nesting environment associated with peak noise measurements.

### 4.4. Covariables Affecting the Onset of Activity

Our results concerning covariables that affect the onset of activity are consistent with previous studies, supporting the reliability of the current study. In accordance with Bruni et al. [54], Naguib et al. [55], Nordt and Klenke [4] and Schlicht and Kempenaers [45], we found a significant delay in the onset of male dawn song and female emergence time following colder nights. The onset of female calling showed the same pattern. As suggested by Schlicht and Kempenaers [45], lower temperatures may decrease foraging success and increase the need for individuals to save energy.

Consistent with previous studies, the earliest onset of male dawn song occurred early in the period of egg-laying, when the female reached her peak fertility, after which it gradually delayed during the following days [45,46,47,52]. Our study is the first to show that the onset of female calling also delayed after the start of the egg-laying phase. We did not find a change in female emergence time during the early days after the start of the egg-laying phase, contrary to Halfwerk et al. [52] and Schlicht et al. [31]. However, the results from Schlicht et al. [31] indicate that a delay in female emergence time mainly occurs during the late stage of the egg-laying phase, which could explain a lack of a delay found in the early days of the egg-laying phase. Although our results suggest that the lowest night temperature and stage of the egg-laying phase are important covariables explaining differences in the onset of activity, their effect sizes are relatively small compared to ALAN. This is contrary to the results obtained by Stuart et al. [90], who found that the onset of male dawn song in House wrens (*Troglodytes aedon*) was mainly influenced by (social) environmental factors and not by daily fluctuations in levels of ALAN and noise pollution.

### 4.5. High Repeatability of the Onset of Activity

Few studies have reported repeatabilities of the onset of male dawn song [55,77,91]. The repeatability in our study is substantially higher than in two other studies in great tits [55,77]. This may be explained by the fact that we (1) studied an urban population with a much larger range in the onset of male dawn song (148 min) due to ALAN and noise pollution compared to the studied Dutch forest population (range 45 min); and (2) measured the onset of male song on successive days in the beginning of the egg-laying period while the two previous studies considered a longer period covering both the early and late stage of egg laying.

As already demonstrated by Meijdam et al. [92], female emergence time in our urban population was also highly repeatable and more repeatable than in female blue tits from a German forest population [31]. Finally, the onset of female calling was only moderately repeatable, which is not surprising given that it is dependent on both male and female behaviour, making the within-individual variance higher. To the best of our knowledge, our study is the first to report the repeatability of the onset of female vocal activity. 

## 5. Conclusions

Consistent with previous studies, we have shown an advancing effect of ALAN on the onset of male dawn song. Furthermore, our study offers a new perspective on the influence of ALAN on female cavity-nesting songbirds during the breeding season. It suggests that, when taking the onset of female calling as a proxy, the urban stressors influencing the female chronotype largely reflect the stressors influencing the chronotype of the male. Field experiments in which exposure to ALAN is alternated between the members of a pair are recommended to further explore this. Noise pollution only affected the onset of activity in females. Finally, the high repeatabilities and the fact that proxies for the chronotype of the male and the female and their interaction have been taken into account in this study make the conclusions that can be drawn from the results more robust.

## Data Availability

All data that support the findings of this study are available at https://doi.org/10.6084/m9.figshare.27285195.v1.

## Supplementary Materials

The following supporting information can be downloaded at https://www.mdpi.com/article/10.3390/ani14223199/s1. Figure S1: Distribution map of the study population; Figure S2: Model assumptions (A: onset of male dawn song; B: female emergence time; C: onset of female calling); Figure S3: Model assumption robust model (A: onset of male dawn song; B: female emergence time; C: onset of female calling); Figure S4: Forest plot regression coefficient predictors; Figure S5: Interaction effect of ALAN and noise pollution on the onset of female calling; Figure S6: Johnson–Neyman interaction plots; Table S1: Number of observations per nest box; Table S2: Model selection linear mixed-effects models (A: onset of male dawn song; B: female emergence time; C: onset of female calling); Table S3: Statistical output mixed-effects models (A: onset of male dawn song; B: female emergence time; C: onset of female calling).
