# Supplementary material for: Artificial Light at Night Advances the Onset of Vocal Activity in Both Male and Female Great Tits During the Breeding Season, While Noise Pollution Has Less Impact and Only in Females
Source: Animals (Basel). 2024 Nov 7;14(22):3199. doi: 10.3390/ani14223199 (PMC11590875; doi:10.3390/ani14223199)
Supplement: Supplementary file 1 [file animals-14-03199-s001.zip › animals-3242418-supplementary.pdf]

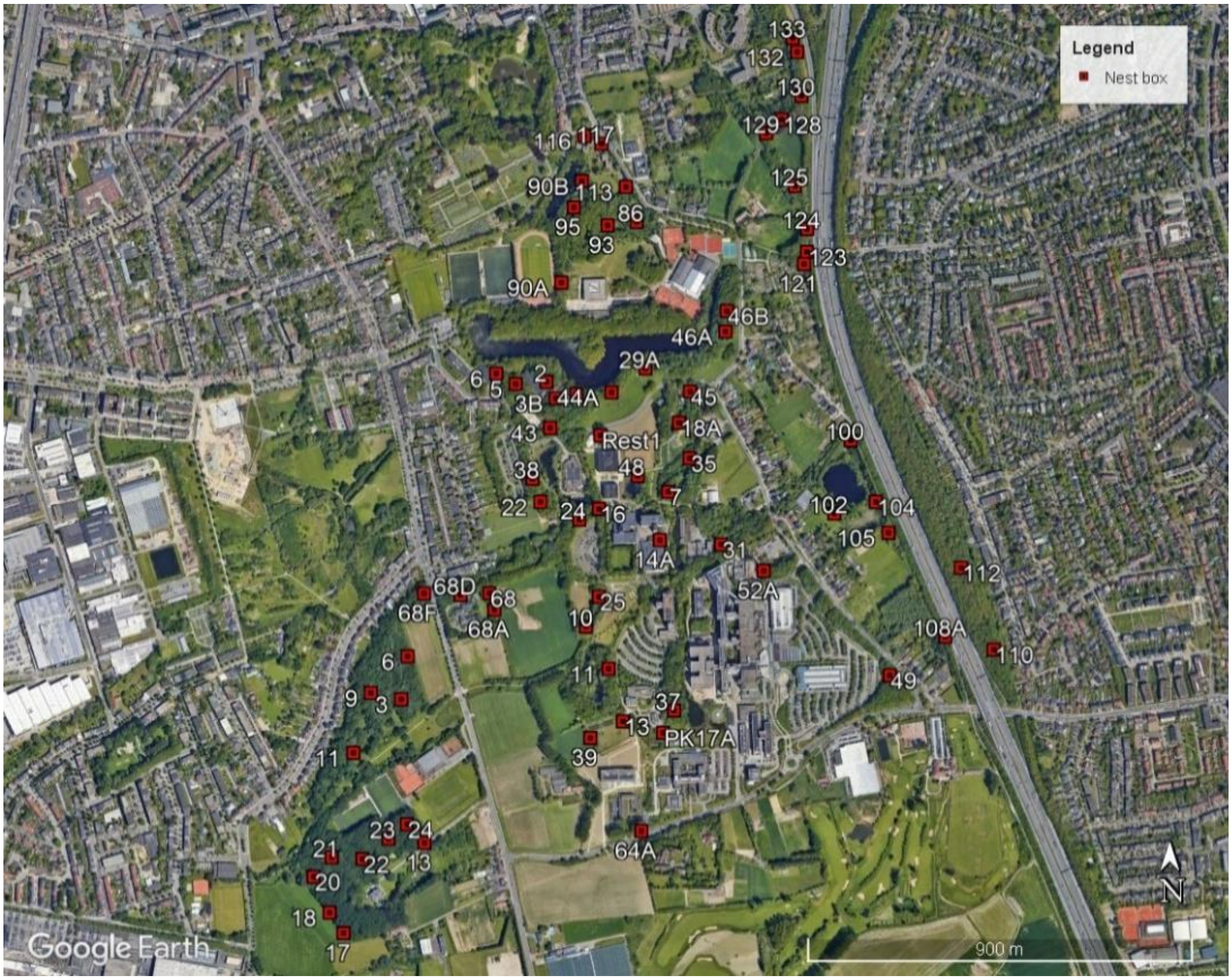

Figure S1. Distribution map of the study population. The map shows the distribution of the nest boxes from the study population at Drie Eiken university campus (Wilrijk, Antwerp) that were used in the analysis.

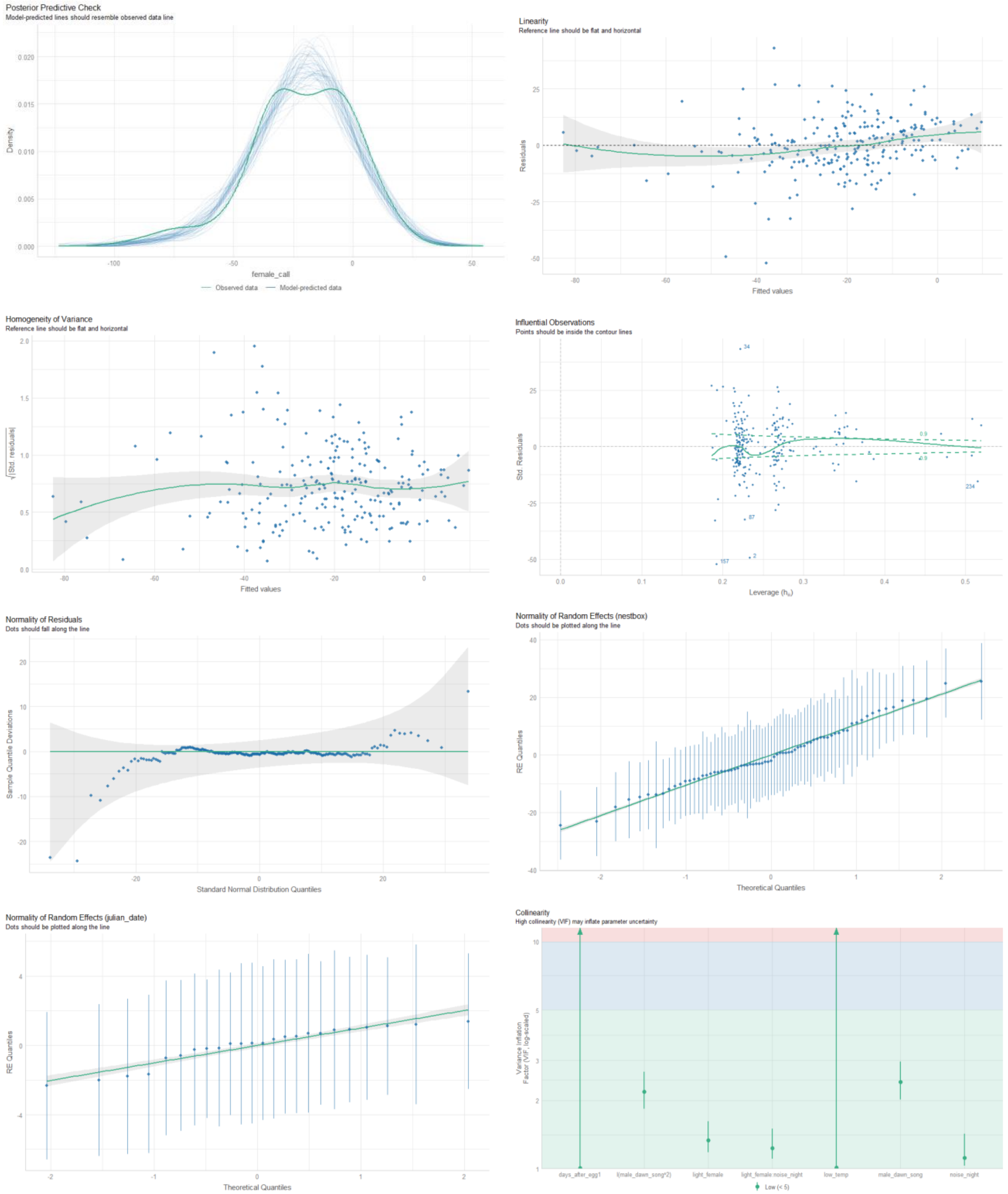

Figure S2A. Model assumptions model onset of male dawn song. Fitted versus residual plots, scale-location plots, the variance inflation factor (VIF), cooks distances and quantile-quantile plots (Q-Q plot) are shown for the model for the onset of male dawn song and were created using the Performance Package [71].

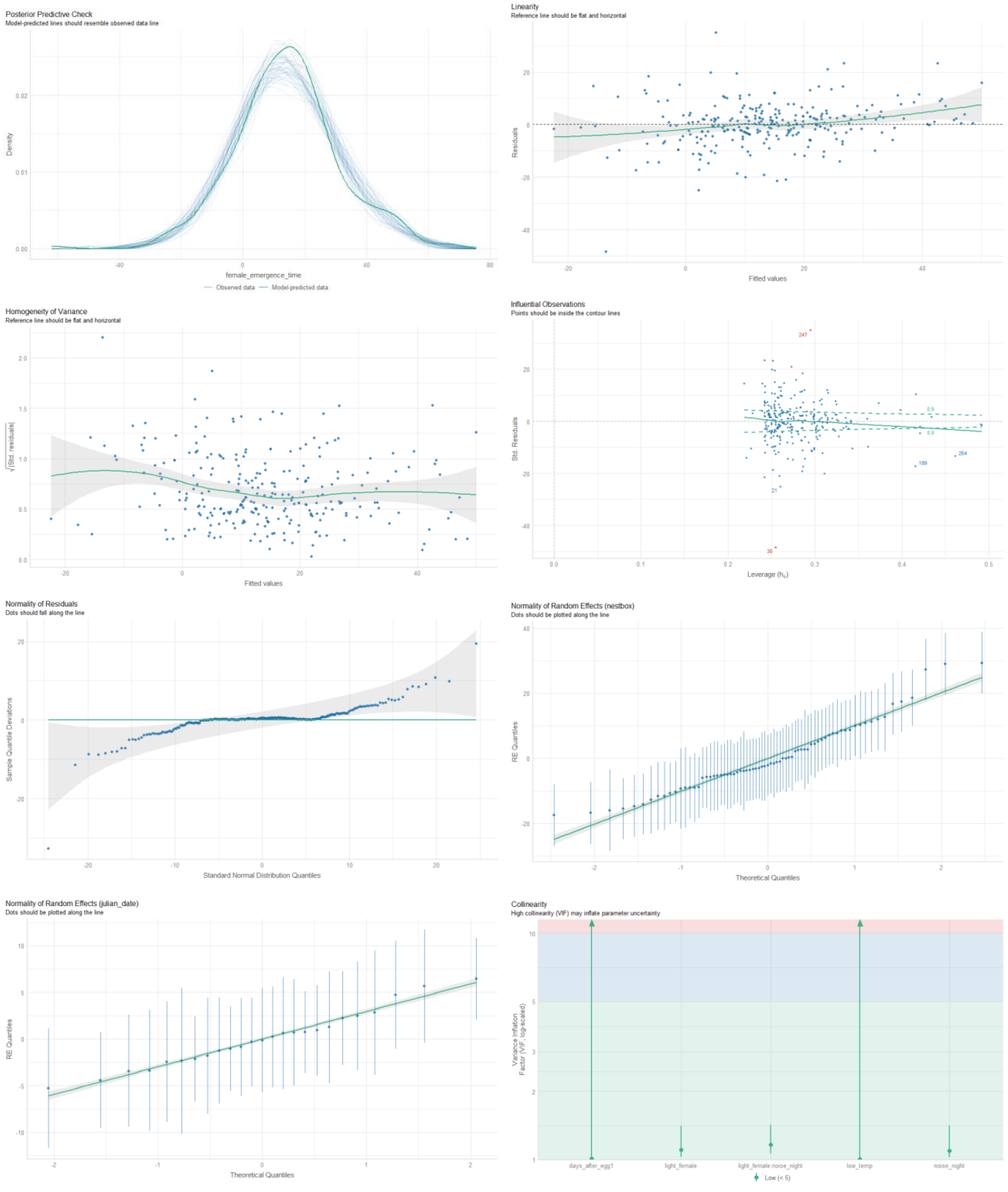

Figure S2B. Model assumptions model female emergence time. Fitted versus residual plots, scale-location plots, the variance inflation factor (VIF), cooks distances and quantile-quantile plots (Q-Q plot) are shown for the model for the female emergence time and were created using the Performance Package [71].

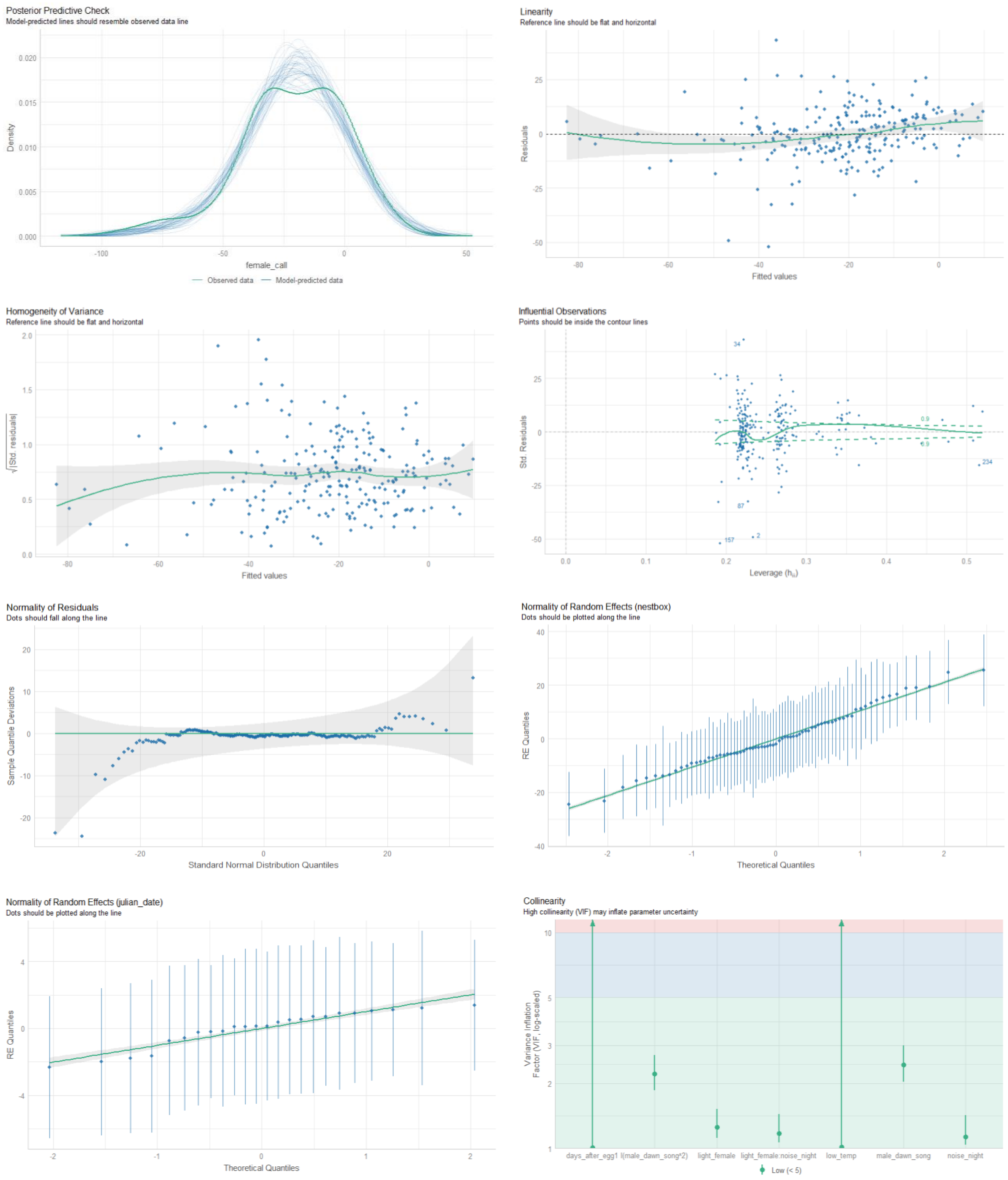

Figure S2C. Model assumptions model onset of female calling. Fitted versus residual plots, scale-location plots, the variance inflation factor (VIF), cooks distances and quantile-quantile plots (Q-Q plot) are shown for the model for the onset of female calling and were created using the Performance Package [71].

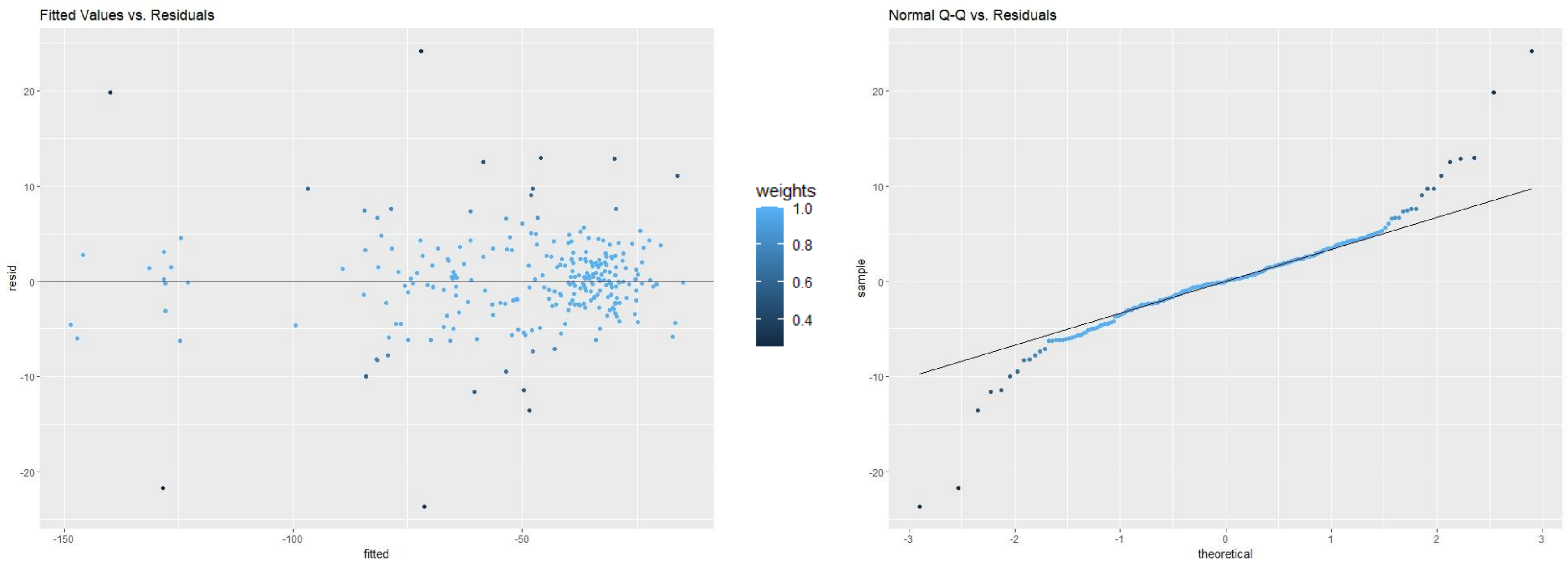

Figure S3A. Model assumptions robust model onset of male dawn song. Fitted versus residual plots and quantile-quantile plots (Q-Q plot) are shown for the model with a robust fit for the onset of male dawn song and were created using the package robustlmm [72].

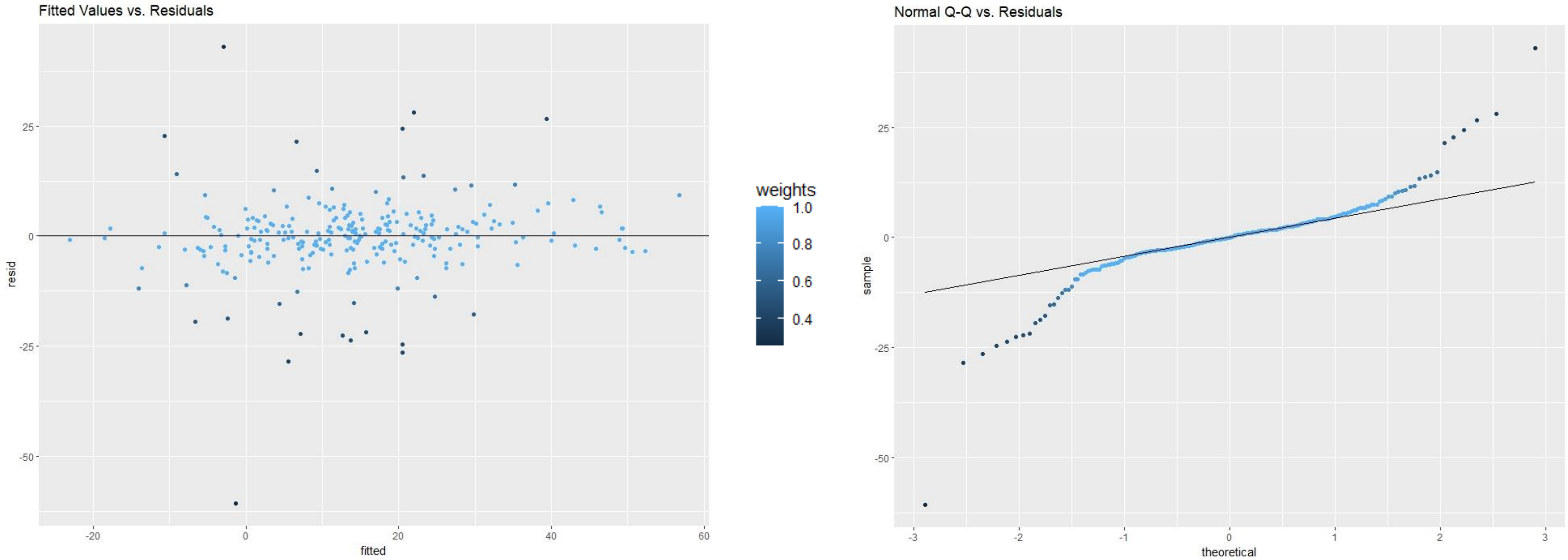

Figure S3B. Model assumptions robust model female emergence time. Fitted versus residual plots and quantile-quantile plots (Q-Q plot) are shown for the model with a robust fit for female emergence time and were created using the package robustlmm [72].

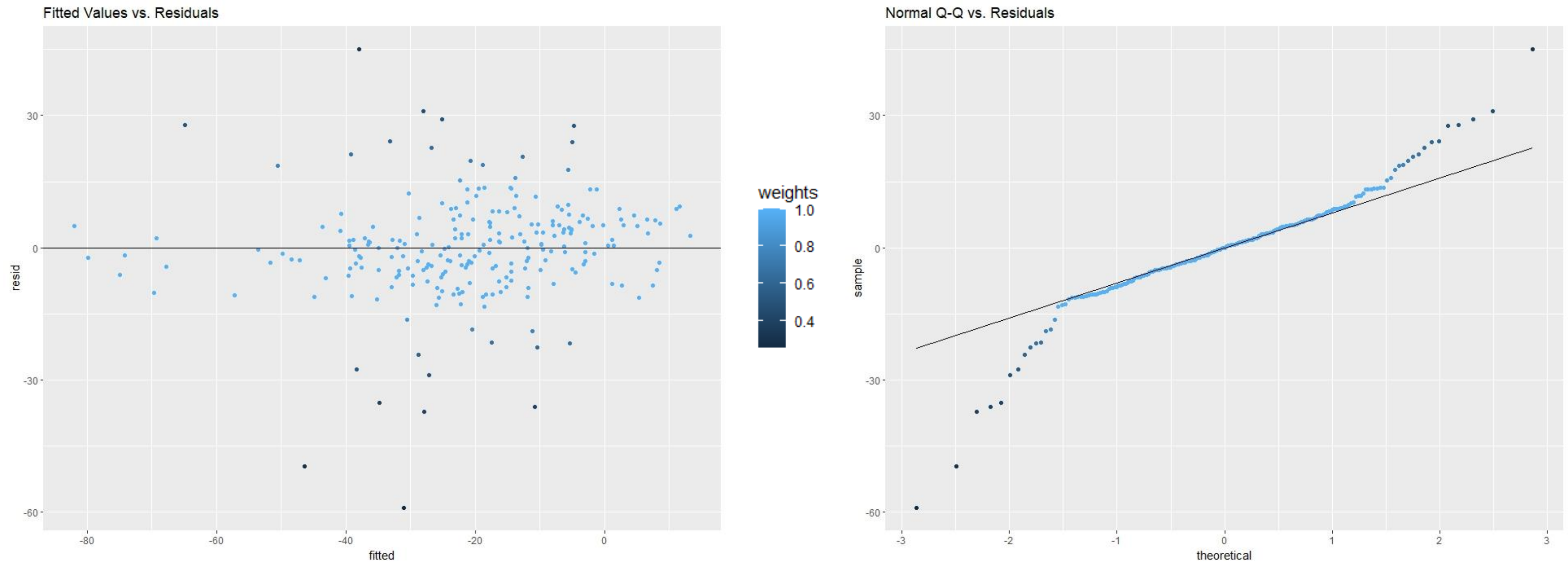

Figure S3C. Model assumptions robust model onset of female calling. Fitted versus residual plots and quantile-quantile plots (Q-Q plot) are shown for the model with a robust fit for the onset of female calling and were created using the package robustlmm [72].

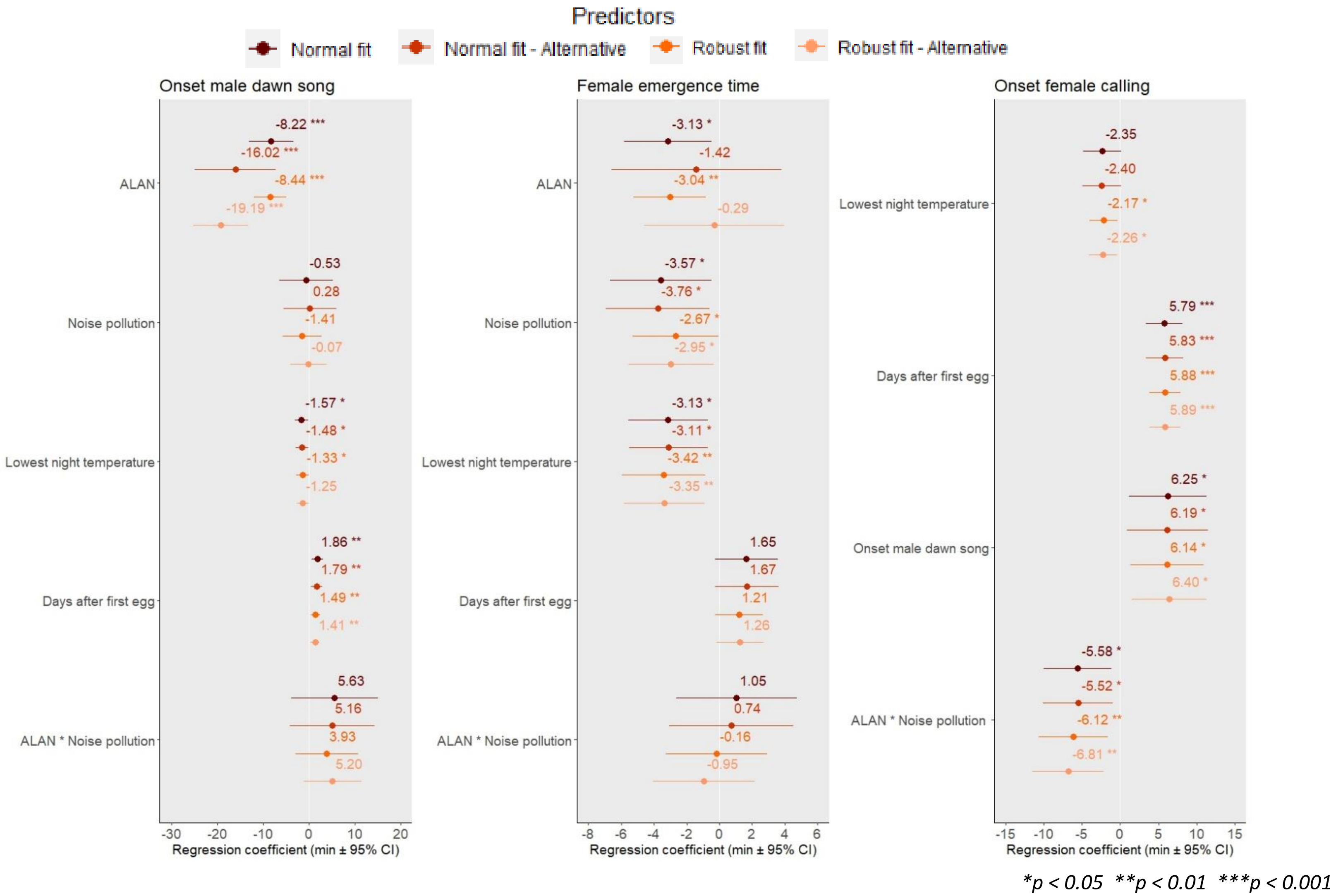

Figure S4. Forest plots regression coefficients predictors. For each onset of activity the regression coefficients for the predictors are shown for all models, including a model with a normal or robust fit and a full or alternative dataset (indicated by the different colours). The coefficients are given in minutes (± 95% CI) and since the predictors are standardized, the coefficients can be compared among the different predictors.

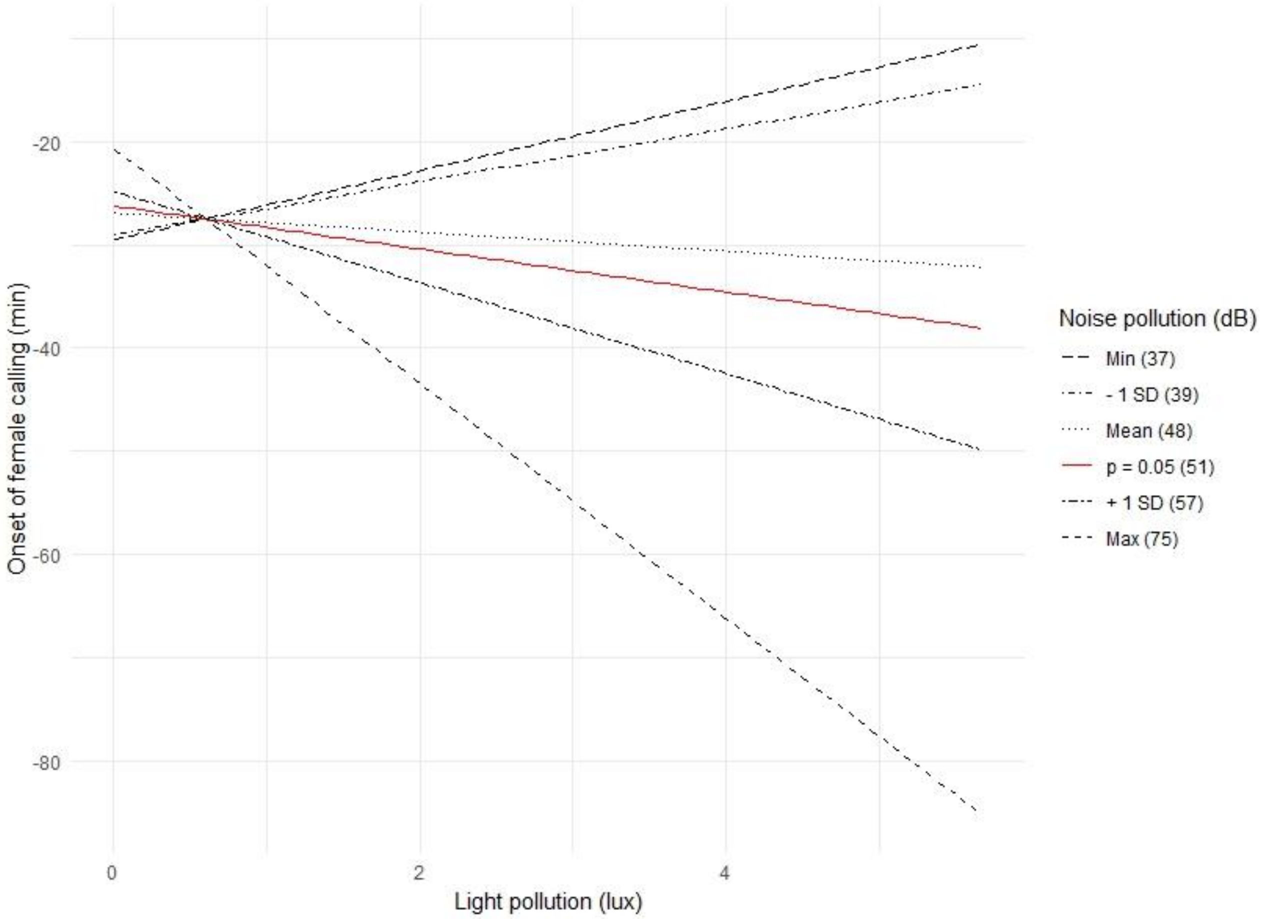

Figure S5. Interaction effect of ALAN (= light pollution) and noise pollution on the onset of female calling. For the effect of ALAN on the onset of female calling predicted regression slopes of the alternative model with a robust fit are shown. The regression slopes for several indicative values of noise pollution are shown for illustrative purposes, including the slope in red for which p = 0.05 (based on Johnson-Neyman plot; Figure S6). Slopes above the red slope do not significantly differ from zero, while slopes below do.

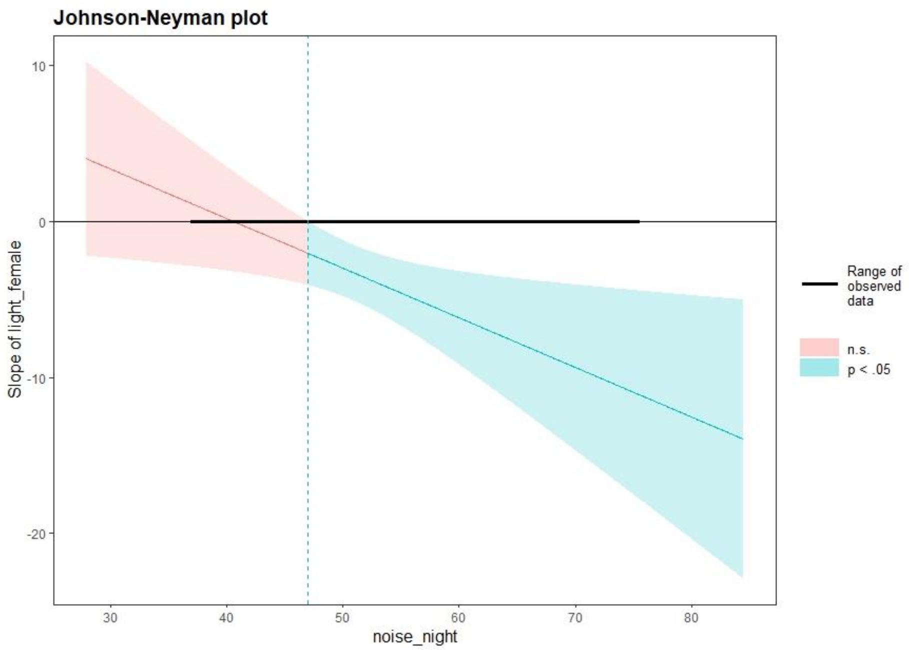

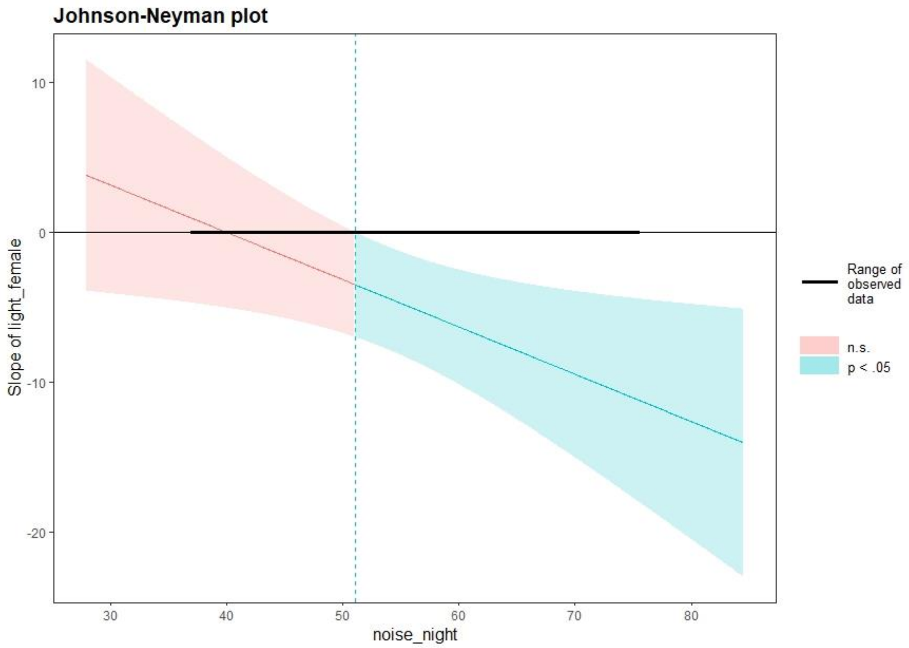

Figure S6. Johnson-Neyman interaction plots. These plot were created using the johnson_neyman() function in the interactions package [70] and indicate the size and significance of the slope of ALAN (= light_female) on the onset of female calling throughout all observed values of noise pollution (= noise_night) for the robust (top) and alternative robust (bottom) fit. The shaded regions indicate 95% CIs.

Table S1. Number of observations per nest box.

A. Number of observations per nest box for the onset of male dawn song[1]	
**Number of observations**	**Amount of individuals/nest boxes**
2	5
3	20
4	47
5	2
	74

B. Number of observations per nest box for female emergence time[1]	
**Number of observations**	**Amount of individuals/nest boxes**
2	5
3	20
4	46
5	2
	73

C. Number of observations per nest box for the onset of female calling[1]	
**Number of observations**	**Amount of individuals/nest boxes**
1	4
2	11
3	23
4	33
5	2
	73

[1] The number of observations per nest box are given, e.g. there are 3 mornings measured for 23 nest boxes (= individuals)

Table S2A. Model Selection linear mixed-effects models onset of male dawn song. This table presents the regression estimates (light_male = ALAN; noise_night = noise pollution; light_male*noise_night = ALAN x noise pollution; low_temp = lowest night temperature; avg_temp_sunrise = mean temperature around sunrise; days_after_egg1 = amount of days after the first egg; avg_prec_sunrise = mean precipitation around sunrise) , degrees of freedom (df), log-likelihood (logLik), AICc, delta AICc (Delta), model weights (weight), and marginal and conditional R^2 for the top three linear mixed-effects models (LMER).

Model	Intercept	light_male	noise_night	light_male* noise_night	low_temp	avg_temp_ sunrise	days_after _egg1	avg_prec_ sunrise	df	logLik	AICc	Delta	Weight	Conditional R^2	Marginal R^2
ModelMaleDawnSong_ Lowtemp_Daysafteregg1 [1]	-50.52	-8.22	-0.53	5.61	-1.58	NA	1.86	NA	9	-1006.36	2031.42	0.00	0.55	0.96	0.12
ModelMaleDawnSong_ Avgtemp_Daysafteregg1 [2]	-50.58	-8.21	-0.54	5.56	NA	-1.23	1.71	NA	9	-1007.25	2033.20	1.78	0.22	0.96	0.12
ModelMaleDawnSong_ Daysafteregg1 [3]	-50.69	-8.23	-0.42	5.57	NA	NA	1.88	NA	8	-1008.54	2033.63	2.21	0.18	0.96	0.11

1. lmer(male_dawn_song ~ light_male + noise_night+ noise_night:light_male + low_temp+ days_after_egg1+ (1|nestbox)+ (1|julian_date) , data= chronotypeDataModel, REML = FALSE, control=lmerControl(optimizer="bobyqa"), na.action = na.fail)
2. lmer(male_dawn_song ~ light_male + noise_night+ noise_night:light_male + avg_temp_sunrise+ days_after_egg1+ (1|nestbox)+ (1|julian_date) , data= chronotypeDataModel, REML = FALSE, control=lmerControl(optimizer="bobyqa"), na.action = na.fail)
3. lmer(male_dawn_song ~ light_male + noise_night+ noise_night:light_male + days_after_egg1+ (1|nestbox)+ (1|julian_date), data= chronotypeDataModel, REML = FALSE, control=lmerControl(optimizer="bobyqa"), na.action = na.fail)

Table S2B. Model Selection linear mixed-effects models female emergence time. This table presents the regression estimates (light_female = ALAN; noise_night = noise pollution; light_female*noise_night = ALAN x noise pollution; low_temp = lowest night temperature; avg_temp_sunrise = mean temperature around sunrise; days_after_egg1 = amount of days after the first egg; avg_prec_sunrise = mean precipitation around sunrise; Male_dawn_song = onset of male dawn song) , degrees of freedom (df), log-likelihood (logLik), AICc, delta AICc (Delta), model weights (weight), and marginal and conditional R^2 for the top three linear mixed-effects models (LMER).

Model	Intercept	light_female	noise_night	light_female* noise_night	low_temp	avg_temp _sunrise	days_after _egg1	avg_prec_ sunrise	Male_ dawn_ song	df	logLik	AICc	Delta	Weight	Conditional R^2	Marginal R^2
ModelFemaleEmergence Time_Lowtemp_Daysafteregg1 [1]	12.87	-3.08	-3.71	1.10	-3.08	NA	1.67	NA	NA	9	-1054.06	2126.83	0.00	0.33	0.64	0.13
ModelFemaleEmergence Time_Lowtemp [2]	12.99	-3.23	-3.59	1.04	-3.24	NA	NA	NA	NA	8	-1055.48	2127.53	0.70	0.23	0.64	0.12
ModelFemaleEmergence Time_Avgtemp [3]	12.86	-3.19	-3.63	0.93	NA	-3.04	NA	NA	NA	8	-1055.75	2128.06	1.22	0.18	0.64	0.11

1. lmer(female_emergence_time ~ light_female + noise_night+ noise_night:light_female + low_temp+ days_after_egg1+ (1|nestbox)+ (1|julian_date), data= chronotypeDataModelF, REML = FALSE, control=lmerControl(optimizer="bobyqa"), na.action = na.fail)
2. lmer(female_emergence_time ~ light_female + noise_night+ noise_night:light_female + low_temp+ (1|nestbox)+ (1|julian_date), data= chronotypeDataModelF, REML = FALSE, control=lmerControl(optimizer="bobyqa"), na.action = na.fail)
3. lmer(female_emergence_time ~ light_female + noise_night+ noise_night:light_female + avg_temp_sunrise+ (1|nestbox)+ (1|julian_date), data= chronotypeDataModelF, REML = FALSE, control=lmerControl(optimizer="bobyqa"), na.action = na.fail)

Table S2C. Model Selection linear mixed-effects models onset of female calling. This table presents the regression estimates (light_female = ALAN; noise_night = noise pollution; light_female*noise_night = ALAN x noise pollution; low_temp = lowest night temperature; avg_temp_sunrise = mean temperature around sunrise; days_after_egg1 = amount of days after the first egg; avg_prec_sunrise = mean precipitation around sunrise; Male_dawn_song = onset of male dawn song) , degrees of freedom (df), log-likelihood (logLik), AICc, delta AICc (Delta), model weights (weight), and marginal and conditional R^2 for the top three linear mixed-effects models (LMER).

Model	Intercept	light_female	noise_night	light_female* noise_night	low_temp	avg_temp _sunrise	days_after _egg1	avg_prec_ sunrise	Male_ dawn_ song	df	logLik	AICc	Delta	Weight	Conditional R^2	Marginal R^2
ModelFemaleCall_Lowtemp_ MaleDS_Daysafteregg1 [1]	-19.33	-5.33	-0.187	-5.76	-2.38	NA	5.94	NA	3.17	10	-1004.82	2030.61	0.00	0.29	0.62	0.28
ModelFemaleCall_Avgtemp_ MaleDS_Daysafteregg1 [2]	-19.30	-5.31	-0.189	-5.86	NA	-2.08	5.56	NA	3.19	10	-1005.26	2031.49	0.88	0.19	0.62	0.28
ModelFemaleCall_Lowtemp_ Daysafteregg1 [3]	-19.49	-6.41	-0.33	-5.15	-2.66	NA	5.97	NA	NA	9	-1006.38	2031.55	0.94	0.18	0.61	0.27

1. llmer(female_call ~ light_female + noise_night+ noise_night:light_female + low_temp+ male_dawn_song+days_after_egg1+ (1|nestbox)+ (1|julian_date), data= chronotypeDataModelFC, REML = FALSE, control=lmerControl(optimizer="bobyqa"), na.action = na.fail)
2. lmer(female_call ~ light_female + noise_night+ noise_night:light_female + avg_temp_sunrise+ male_dawn_song+days_after_egg1+ (1|nestbox)+ (1|julian_date), data= chronotypeDataModelFC, REML = FALSE, control=lmerControl(optimizer="bobyqa"), na.action = na.fail)
3. lmer(female_call ~ light_female + noise_night+ noise_night:light_female + low_temp+ days_after_egg1+ (1|nestbox)+ (1|julian_date), data= chronotypeDataModelFC, REML = FALSE, control=lmerControl(optimizer="bobyqa"), na.action = na.fail)

Table S3A. Statistical output mixed effect models onset of male dawn song. For the model with a normal fit and alternative models with a normal and robust fit, the fixed effect part of the table shows the regression estimates (with standard error) in minutes, 95% confidence intervals (CI 95%) , t-values and p-values (Satterthwaite approximation). All predictors are standardized, meaning that for each standard deviation increase in the predictor the change in the onset of activity (in minutes) is given by the estimate. Significant values ($p < 0.05$) are depicted in bold. In the random effect part of the table the variance of both random factors is shown (τ00), as well as the residual variance (σ^2) and the intraclass correlation coefficient (ICC). The marginal and conditional R^2 for the robust mixed effect models are calculated using the robustlmm package [72] according to Nakagawa & Schielzeth [74].

	Model onset male dawn song					*Alternative model onset male dawn song*					*Robust alternative model onset male dawn song*				
**Predictors**	**Estimates**	**CI (95%)**	**t-value**	**p-value**	**df**	**Estimates**	**CI (95%)**	**t-value**	**p-value**	**df**	**Estimates**	**CI (95%)**	**t-value**	**p-value**	**df**
(Intercept)	-50.54 (3.03)	-56.57 – -44.51	-16.7	**<0.001**	73.59	-51.43 (3.00)	-57.40 – -45.46	-17.16	**<0.001**	72.67	-48.51 (2.08)	-52.58 – -44.44	-23.37	**<0.001**	NA
ALAN	-8.22 (2.47)	-13.14 – -3.30	-3.33	**0.001**	70.99	-16.02 (4.53)	-25.04 – -6.99	-3.54	**<0.001**	69.03	-19.19 (3.08)	-25.24 – -13.15	-6.22	**<0.001**	NA
Noise pollution	-0.53 (2.98)	-6.48 – 5.42	-0.18	0.86	69.96	0.28 (2.95)	-5.60 – 6.16	0.09	0.925	68.95	-0.07 (2.01)	-4.01 – 3.86	-0.04	0.925	NA
Lowest night temperature	-1.57 (0.74)	-3.10 – -0.04	-2.11	**0.045**	25.6	-1.48 (0.75)	-3.01 – 0.05	-1.99	0.058	25.79	-1.25 (0.68)	-2.57 – 0.07	-1.85	0.058	NA
Days after first egg	1.86 (0.63)	0.61 – 3.11	2.94	**0.003**	187.3	1.79 (0.63)	0.55 – 3.03	2.84	**0.005**	186.5	1.41 (0.52)	0.38 – 2.43	2.69	**0.005**	NA
*ALAN * Noise pollution*	5.63 (4.79)	-3.93 – 15.20	1.18	0.244	70.08	5.16 (4.70)	-4.21 – 14.52	1.1	0.276	69.1	5.20 (3.20)	-1.07 – 11.48	1.62	0.276	NA
**Random effects**															
σ²	31.1					30.62					19.67				
τ00 nest box ID	626.69					599.65					262.55				
τ00 Julian date	5.78					5.90					5.57				
ICC	0.95					0.95					0.93				
N nest box ID	74					73					73				
N Julian date	26					26					26				
Observations	268					266					266				
Marginal R² / Conditional R²	0.11 / 0.96					0.16 / 0.96					0.37 / 0.96				

Table S3B. Statistical output mixed effect models female emergence time**.** For the model with a normal fit and alternative models with a normal and robust fit, the fixed effect part of the table shows the regression estimates (with standard error) in minutes, 95% confidence intervals (CI 95%) , t-values and p-values (Satterthwaite approximation). All predictors are standardized, meaning that for each standard deviation increase in the predictor the change in the onset of activity (in minutes) is given by the estimate. Significant values ($p < 0.05$) are depicted in bold. In the random effect part of the table the variance of both random factors is shown (τ00), as well as the residual variance (σ^2) and the intraclass correlation coefficient (ICC). The marginal and conditional R^2 for the robust mixed effect models are calculated using the robustlmm package [72] according to Nakagawa & Schielzeth [74].

	Model female emergence time					*Alternative model female emergence time*					*Robust alternative model female emergence time*				
**Predictors**	**Estimates**	**CI (95%)**	**t-value**	**p-value**	**df**	**Estimates**	**CI (95%)**	**t-value**	**p-value**	**df**	**Estimates**	**CI (95%)**	**t-value**	**p-value**	**df**
(Intercept)	12.99 (1.80)	9.38 – 16.60	7.2	**<0.001**	58.92	13.24 (1.83)	9.58 – 16.89	7.24	**<0.001**	59.93	13.05 (1.72)	9.67 – 16.43	7.57	**<0.001**	NA
ALAN	-3.13 (1.36)	-5.84 – -0.42	-2.3	**0.024**	81.32	-1.42 (2.64)	-6.68 – 3.85	-0.54	0.593	67.78	-0.29 (2.18)	-4.56 – 3.97	-0.14	0.593	NA
Noise pollution	-3.57 (1.58)	-6.72 – -0.41	-2.26	**0.027**	68.09	-3.76 (1.61)	-6.97 – -0.54	-2.33	**0.023**	66.96	-2.95 (1.32)	-5.55 – -0.36	-2.23	**0.023**	NA
Lowest night temperature	-3.13 (1.24)	-5.68 – -0.57	-2.52	**0.022**	25.6	-3.11 (1.23)	-5.64 – -0.58	-2.52	**0.021**	25.79	-3.35 (1.25)	-5.80 – -0.89	-2.67	**0.021**	NA
Days after first egg	1.65 (0.99)	-0.31 – 3.61	1.66	0.094	230.83	1.67 (1.00)	-0.29 – 3.64	1.68	0.089	228.87	1.26 (0.74)	-0.19 – 2.72	1.71	0.089	NA
*ALAN * Noise pollution*	1.05 (1.87)	-2.67 – 4.77	0.56	0.575	70.24	0.74 (1.92)	-3.09 – 4.57	0.38	0.702	68.52	-0.95 (1.58)	-4.05 – 2.15	-0.6	0.575	NA
**Random effects**															
σ²	99.99					100.69					41.55				
τ00 nest box ID	136.55					138.19					95.43				
τ00 Julian date	17.61					16.78					26.23				
ICC	0.61					0.61					0.75				
N nest box ID	73					72					72				
N Julian date	25					25					25				
Observations	264					262					262				
Marginal R² / Conditional R²	0.12 / 0.66					0.09 / 0.64					0.12 / 0.78				

Table S3C. Statistical output mixed effect models onset of female calling. For the model with a normal fit and alternative models with a normal and robust fit, the fixed effect part of the table shows the regression estimates (with standard error) in minutes, 95% confidence intervals (CI 95%) , t-values and p-values (Satterthwaite approximation). All predictors are standardized, meaning that for each standard deviation increase in the predictor the change in the onset of activity (in minutes) is given by the estimate. Significant values ($p < 0.05$) are depicted in bold. In the random effect part of the table the variance of both random factors is shown ($\tau 00$), as well as the residual variance (σ^2) and the intraclass correlation coefficient (ICC). The marginal and conditional R^2 for the robust mixed effect models are calculated using the robustlmm package [72] according to Nakagawa & Schielzeth [74].

	Model onset female calling					*Alternative model onset female calling*					*Robust alternative model onset female calling*				
**Predictors**	**Estimates**	**CI (95%)**	**t-value**	**p-value**	**df**	**Estimates**	**CI (95%)**	**t-value**	**p-value**	**df**	**Estimates**	**CI (95%)**	**t-value**	**p-value**	**df**
(Intercept)	-21.55 (2.22)	-25.99 – -17.11	-9.69	**<0.001**	64.67	-21.56 (2.27)	-26.09 – -17.03	-9.51	**<0.001**	63.6	-20.53 (2.21)	-24.85 – -16.20	-9.3	**<0.001**	NA
ALAN	-4.79 (1.85)	-8.46 – -1.12	-2.59	**0.011**	82.93	-5.13 (3.59)	-12.29 – 2.03	-1.43	0.156	66.3	-2.04 (3.64)	-9.19 – 5.10	-0.56	0.156	NA
Noise pollution	0.10 (1.85)	-3.58 – 3.78	0.05	0.956	70.7	0.13 (1.88)	-3.63 – 3.88	0.07	0.947	69.1	-0.15 (1.91)	-3.89 – 3.60	-0.08	0.947	NA
Lowest night temperature	-2.35 (1.31)	-5.05 – 0.34	-1.8	0.086	24.4	-2.40 (1.31)	-5.10 – 0.30	-1.83	0.081	24.7	-2.26 (0.96)	-4.14 – -0.38	-2.36	0.081	NA
Days after first egg	5.79 (1.25)	3.33 – 8.25	4.64	**<0.001**	201.3	5.83 (1.25)	3.35 – 8.30	4.65	**<0.001**	200	5.89 (1.03)	3.86 – 7.91	5.7	**<0.001**	NA
Onset male dawn song	6.25 (2.62)	1.06 – 11.44	2.38	**0.017**	118.2	6.19 (2.72)	0.81 – 11.57	2.28	**0.022**	120	6.40 (2.50)	1.50 – 11.29	2.56	**0.022**	NA
Onset male dawn song²	1.78 (1.08)	-0.36 – 3.93	1.64	0.098	125.6	1.75 (1.09)	-0.42 – 3.91	1.6	0.108	125	1.75 (1.01)	-0.24 – 3.73	1.73	0.108	NA
*ALAN * Noise pollution*	-5.58 (2.24)	-10.04 – -1.11	-2.49	**0.015**	66.33	-5.52 (2.33)	-10.17 – -0.87	-2.37	**0.021**	64.7	-6.81 (2.38)	-11.47 – -2.14	-2.86	**0.021**	NA
**Random effects**															
σ²	187.15					188.28					115.21				
τ00 nest box ID	166.83					169.88					194.68				
τ00 Julian date	6.12					5.77					0.00				
ICC	0.48					0.48					0.63				
N nest box ID	73					72					72				
N Julian date	24					24					24				
Observations	237					235					235				
Marginal R² / Conditional R²	0.28 / 0.63					0.24 / 0.61					0.26 / 0.72				
